# Bidirectional relationship between serum creatinine to cystatin C ratio and chronic kidney disease: a mediation analysis of depression in a national aging cohort

**DOI:** 10.3389/fpsyt.2025.1554695

**Published:** 2025-05-16

**Authors:** Qiong Cai, Qing Zhong, Sajid Ali, Wenting Yang, Yanan Chen, Bo Ao, Youliang Huang

**Affiliations:** ^1^ Department of Social Medicine and Health Education, School of Public Health, Peking University, Beijing, China; ^2^ School of Management, Beijing University of Chinese Medicine, Beijing, China; ^3^ First Affiliated Hospital & Clinical Medical College, Gannan Medical University, Ganzhou, Jiangxi, China; ^4^ Department of Information Sciences, University of Education, Multan, Pakistan; ^5^ Department of Acupuncture, Beijing Tongzhou District Hospital of Traditional Chinese Medicine Integrated Western Medicine, Beijing, China; ^6^ Department of Pediatric, China-Japan Friendship Hospital, Beijing, China; ^7^ National Institute of Chinese Medicine Development and Strategy, Beijing University of Chinese Medicine, Beijing, China

**Keywords:** serum creatinine to cystatin C ratio, depressive symptoms, chronic kidney disease, all-cause mortality, mediating effect

## Abstract

**Objective:**

CCR is an emerging biomarker for renal function, yet its relationship with depression and CKD risk has not been confirmed and is understudied in the population. This study aimed to investigate the association between CCR and CKD, as well as all-cause mortality, among middle-aged and elderly Chinese individuals. Additionally, it sought to explore the bidirectional mediating effect of depression in the association of CCR with CKD.

**Method:**

This study analyzed participant data from the China Health and Retirement Longitudinal Study(CHARLS) from 2011 to 2020. The 10-item Center for Epidemiological Survey Depression Scale(CES-D-10) was used to assess depressive symptoms. Kaplan-Meier survival analyses were used to generate survival curves for participants stratified by different levels of CCR. Multivariate logistic regression was employed to examine the CCR-CKD, CCR-depression, and CKD-depression association. Multivariate Cox regression assessed the association between CCR and all-cause mortality. The potential mediating effect of depression between CCR and CKD was determined by bidirectional mediation analysis.

**Result:**

The study included a total of 6,243 participants, comprising 2,835 men and 3,408 women. Multivariate logistic regression analysis revealed that CCR was positively correlated with an increased risk of CKD(*OR:1.13, 95%CI:1.09-1.18, p<0.001*) and negatively correlated with the severity of depression(*OR:0.94, 95%CI:0.91-0.96, p<0.001*). Higher CCR levels correlated with lower all-cause mortality(*HR:0.83, 95%CI:0.74-0.97, p<0.001*). Depression had a partial negative mediating effect in the association between CCR and CKD. Restricted cubic spline curves showed a U-shaped, nonlinear association between CCR and all-cause mortality.

**Conclusions:**

Higher levels of CCR are associated with a higher risk of CKD and with lower all-cause mortality, and CCR could be a biomarker for early diagnosis of CKD. Depression may have a negative effect on the association of CKD and CCR, suggesting that CCR has an underestimated risk for predicting and recognizing CKD in depressed populations. Mental health factors are important in risk prediction and comprehensive management of CKD.

## 1 Introduction

Chronic Kidney Disease(CKD) is highly prevalent yet often under-recognized (1), posing a significant threat to human health alongside cardiovascular disease, cancer, diabetes, and chronic obstructive pulmonary disease (2, 3). The pathogenesis of CKD is complex and multifaceted, involving not only elevated glomerular pressure leading to hyperfiltration but also processes such as chronic inflammation, fibrosis, and oxidative stress-induced loss of renal units (4, 5), all of which contribute to irreversible renal function impairment. Early-stage CKD progresses gradually and is challenging to detect, often necessitating prognostication and diagnosis through laboratory test indicators. In addition, CKD has a poor prognosis and is strongly associated with elevated mortality rates in the elderly population (6).

Kidney Disease: Improving Global Outcomes (KDIGO) CKD Work Group defines CKD as persistent abnormalities of renal structure or function for more than 3 months, as evidenced by estimated glomerular filtration rate (eGFR) or the presence of markers of renal injury, changes in urinary sediment, or proteinuria (7). The eGFR is the main form of reflection of changes in renal function, but due to its time-consuming measurement, it is usually estimated using endogenous markers such as serum creatinine and cystatin C (8). Serum creatinine used to be the gold standard for testing renal function, but it is susceptible to interference from other factors thereby affecting the determination of abnormal renal function. Cystatin C is a low molecular protein that is independent of gender, age, and body size, and despite its high sensitivity to early renal abnormalities (9), it is still susceptible to bias in the eGFR. Studies have shown that the combination of the two can accurately predict and improve the progression of kidney disease (8, 10). A meta-analysis indicated that creatinine to Cystatin C Ratio (CCR) can be used to make up for the limitations of a single evaluation index, and can better reduce the influence of other factors on CKD (11), and can assess the occurrence of CKD more comprehensively. Studies have shown that eGFR calculated based on CCR helps to recognize the decline of renal function at an early stage and is predictive of renal failure (1). Early detection of abnormalities can help in the diagnosis and treatment of CKD, and the association between CCR and CKD may provide new perspectives for the early monitoring of CKD. However, most of the current evidence focuses on the association between CCR and CKD mortality, and the association between CCR and CKD has not been established. In addition, higher CCR is associated with a lower risk of death, which we validated (12, 13).

Depression is a complex disorder influenced by the dynamic interplay of genetic characteristics, clinical presentation, environmental exposures, co-morbidities, and psychosocial risk factors, and usually results in a more severe disease burden and significantly impaired quality of life (14). Depression is more common in patients with CKD (15, 16), with a prevalence rate of more than 25%, which is higher than that of the general population (17). Depression was associated with the risk of rapid decline in renal function (*OR:1.15, 95% CI:1.03-1.28*), and more significantly with high depressive symptoms (*OR:1.39, 95% CI:1.03-1.88*) *(* 18). Psychological factors influence the course of CKD to a greater extent, and most of the past studies have focused on the relationship between depression and CKD, ignoring the possibility of depression as a mediator to influence biomarkers and disease development mechanisms. Previous studies have examined the relationship between CCR and depression, with lower CCR associated with depression in men (*OR:0.49, 95%CI:0.31-0.75, p=0.001*) *(* 19). A cross-sectional investigation found that cystatin C may be involved in pathophysiologic processes in depressed patients (20, 21), and may be associated with the promotion of inflammatory factor release or apoptosis (22, 23). Serum creatinine reflects muscle mass, and lower muscle mass affecting social activity is also associated with an increased risk of depression. While existing studies have revealed two-by-two associations of CCR-depression and depression-CKD (18, 19), the mediating effect of depression has not been elucidated. Given the above studies, we tentatively hypothesized a mediating effect of depression between CCR and CKD.

This study aims to examine the role of the CCR as a predictor of CKD and all-cause mortality in a middle-aged and elderly Chinese cohort, evaluating depression as a potential mediator of this association.

## 2 Material and method

### 2.1 Data source and research people

The China Health and Retirement Longitudinal Study ( *https://charls.pku.edu.cn/* ) (CHARLS) is a prospective national cohort study encompassing Chinese rural and urban residents aged 45 years and older. This study is representative of middle-aged and older adults across 150 districts and counties and 450 villages in China, with data collected at five intervals: 2011, 2013, 2015, 2018, and 2020 (24). The CHARLS study was approved by the Biomedical Ethics Committee of Peking University(IRB00001052-11015), and all participants provided written informed consent.

**Figure 1:**
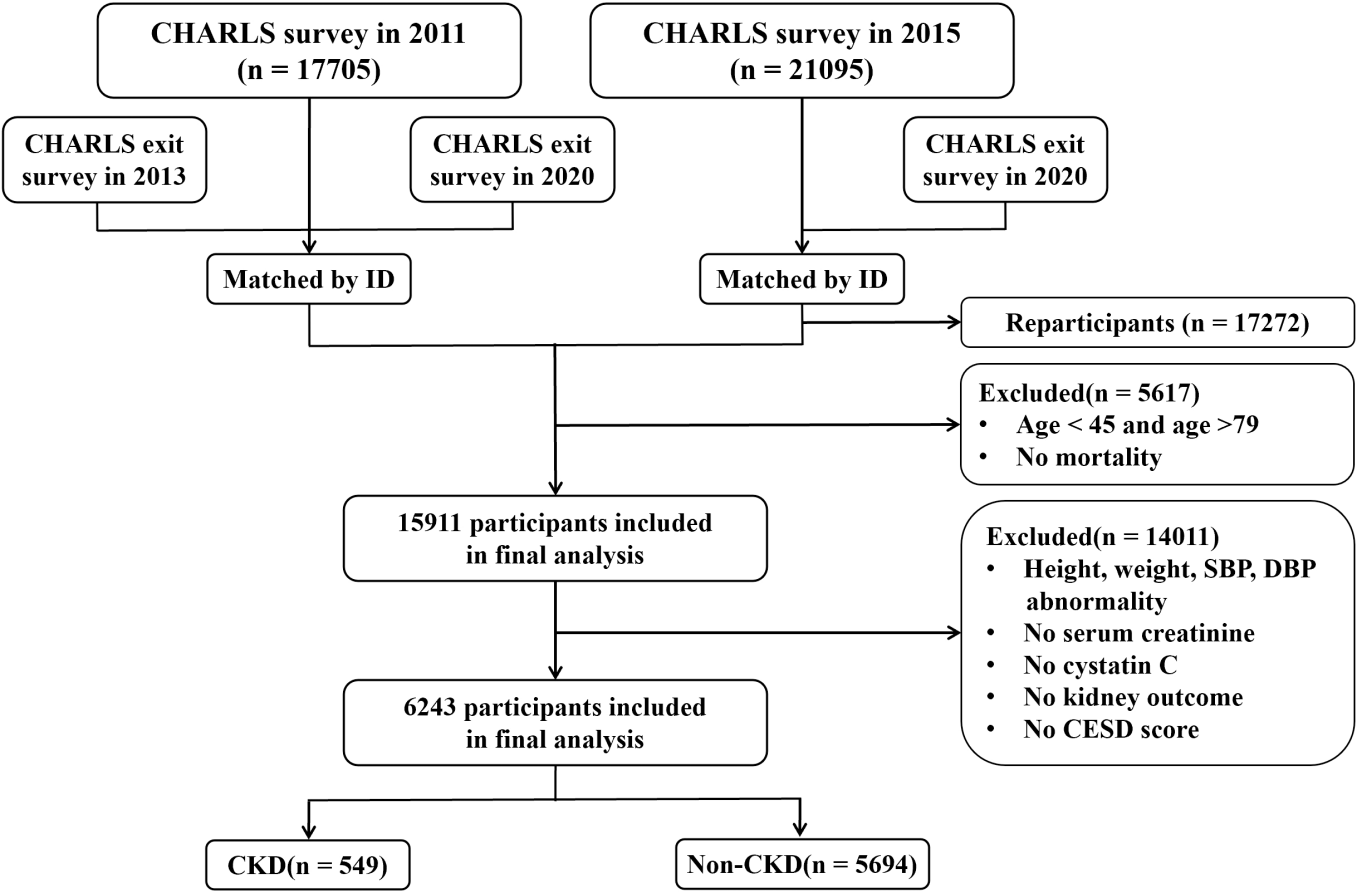
Presents the workflow diagram (CHARLS, China Health and Retirement Longitudinal Study; CESD, Center for Epidemiologic Studies Depression Scale; CKD, Chronic Kidney Disease).

This study included data from the CHARLS spanning from 2011 through 2020. Initially, 17,705 participants completed the baseline data collection during the first survey conducted in 2011, with a subsequent follow-up survey commencing in 2013. Given that blood tests for participants were conducted exclusively in 2011 and 2015, and survival data were collected in 2013 and 2020, the inclusion criteria for this study were as follows: (1) participants who took part in the survey in 2011 and had documented survival status in either 2013 or 2020; (2) participants newly enrolled in 2015 with documented survival status in 2020; (3) participants aged between 45 and 79 years. The exclusion criteria comprised: (1) participants with incomplete data concerning CKD, serum creatinine, cystatin C, depressive symptom scores, and survival status; (2) participants with abnormal values for height(greater than 300 centimeters or less than 100 centimeters), body weight(greater than 200 kilograms), systolic blood pressure(greater than 300 mm Hg), and diastolic blood pressure (greater than 200 mm Hg). Ultimately, a total of 6,243 participants were enrolled in the study to assess the association of baseline CCR levels with the risk of CKD, depression, and all-cause mortality, as well as the bidirectional mediating effect of depression. The study process is shown in **Figure 1**.

### 2.2 Definition of serum creatinine to cystatin C ratio

The CCR index is determined by calculating the ratio of creatinine to cystatin C (25). Participants were instructed to provide blood samples at their local hospital or CDC following a face-to-face interview. These samples were subsequently analyzed to test complete blood counts, as well as levels of serum creatinine, cystatin C, and other blood biomarkers. Notably, serum creatinine is measured in mg/dL and cystatin C is measured in mg/L. To standardize the ratio unit to 1, the serum creatinine unit was converted to mg/L. In this study, participants were categorized into four groups (Q1, Q2, Q3, and Q4) based on the quartiles of the CCR index, with the Q1 group serving as the reference group. For more detailed information on blood sample collection and analysis, please see other topics (26).

### 2.3 Chronic kidney disease events and all-cause mortality measures

The outcome variables for this study were defined as CKD and all-cause mortality. The incidence of CKD was determined through self-reports, wherein a physician inquired, “Have you been diagnosed with Kidney disease(except for tumor or cancer)?” or “Are you now taking any of the following treatments to treat Kidney or its complications(Check all that apply)? Taking Chinese traditional medicine, taking Western modern medicine, other treatments?”. Data based on self-reporting may be subject to selective bias or measurement bias, so there may be some uncertainty in this definition. Additionally, the eGFR is calculated using the CKD epidemiology collaborative equation incorporating both creatinine and cystatin C values. This calculation is instrumental in establishing a definitive diagnosis of CKD (27). An eGFR of less than 60 ml/min/1.73 m^2^ is also considered to be an indication of CKD (28, 29). Although eGFR values may not accurately reflect the true state of renal function in all individuals, it is a standardized measure commonly used in current epidemiologic studies. All-cause mortality was defined as death from any cause within a specified timeframe, with the exact date of death documented on a death certificate or within a medical record. Data collection in the CHARLS database was conducted exclusively in the years 2013 and 2020.

### 2.4 Assessment of depressive symptoms

Depression were assessed using the 10-item Center for Epidemiological Survey Depression Scale(CES-D-10) was used to assess depressive symptoms, which is an adaptation of the original 20-item scale developed by Radloff at the National Institute of Mental Health(USA) (30). This scale has been validated for use among middle-aged and elderly populations in China (31). The CES-D-10 short scale consists of the following items: ① I was bothered by things that usually don’t bother me; ① I had trouble keeping my mind on what I was doing; ③ I felt depressed; ④ I felt that everything I did was an effort; ⑤ I felt hopeful about the future(reverse scoring); ⑥ I felt fearful; ⑦ My sleep was restless; ⑧ I was happy(reverse scoring); ⑨ I felt lonely; ⑩ I could not “get going”. All options are divided into 4 levels: 0 indicates “rarely or not at all”; 1 indicates “not too much”; 2 indicates “sometimes or half the time”; 3 indicates “most of the time”. The total score ranges from 0 to 30, with higher scores reflecting greater depressive symptoms. To categorize CES-D-10 scores and evaluate depressive symptomatology, a threshold value of 10 was employed, where scores below 10 were classified as normal and scores of 10 or higher were indicative of depression (31).

### 2.5 Covariates

In light of the study’s characteristics and previous research, a series of controls were incorporated for sociodemographic, lifestyle, and health status variables (32). Socio-demographic variables included age(both continuous and categorical, with categories: 45-49, 50-59, 60-69, 70-79), gender(female, male) and body mass index(BMI). Lifestyle variables encompassed alcohol consumption(yes, no) and smoking status(smoker, ex-smoker, non-smoker). Health status variables comprised hypertension(yes, no), diabetes mellitus(yes, no), and dyslipidemia(yes, no). BMI was calculated as weight in kilograms divided by height in meters squared(kg/m2). Detailed definitions and categorizations of covariates are provided in the **Supplementary File**, see **Supplementary Table S1** for details.

### 2.6 Handling of missing variables

**Supplementary Figure S1** and **Supplementary Figure S2** show the distribution of data and the missingness of the variables included in our study. The sample has a small proportion of missing data, assuming random missingness. Therefore, we used the method of Multiple Interpolation of Chained Equations (‘MICE’) to deal with the missing data, keeping the sample size as large as possible.

### 2.7 Statistical analyses

This study was statistically analyzed using STATA/MP(version 17, StataCorp LLC, College Station, TX, USA) and R(version 4.4.1, R Core Team, Vienna, Austria) software. Baseline characteristics were stratified according to quartiles of the CCR. Continuous variables that conformed to a normal distribution were presented as mean ± standard deviation, while categorical variables were reported as frequencies(percentage, %). One-way ANOVA was used to compare the characteristics of continuous variables across participants in different CCR quartiles and between participants in CKD and non-CKD. In contrast, the chi-square test was utilized to examine differences in categorical variables among various CCR quartiles, and between participants in CKD and Non-CKD. Kaplan-Meier survival analyses were conducted to generate overall survival curves for CCR across the entire cohort and specifically within the CKD population. Linear regression and multivariate logistic regression models were applied to evaluate odds ratios (ORs) and 95% confidence intervals(95%CI) for the association between CCR and both depressive symptoms and the risk of CKD. Additionally, multivariate Cox regression models were used to assess hazard ratios(HRs) and 95% confidence intervals(95%CI) for the association between CCR and all-cause mortality. Model 1 focused on analyzing the fundamental effect of CCR on the risk of CKD, depressive symptoms, and all-cause mortality, treating CCR or CKD as the independent variable. Additionally, in Model 1, the relationship between depressive and CCR, depression and CKD was examined, with depressive serving as the independent variable. Model 2 expanded upon Model 1 by incorporating sociodemographic variables, including age(as a continuous variable), gender, and BMI. Model 3 further adjusted Model 2 by including individual lifestyle characteristics, specifically alcohol consumption and smoking status. Model 4 extended Model 3 by considering additional health status characteristics, namely hypertension, diabetes, and dyslipidemia.

To investigate potential linear or nonlinear relationships between CCR indices, depressive symptoms, renal disease incidence, and all-cause mortality, restricted cubic spline(RCS) linear regression was applied to Model 4 to mitigate the influence of outliers. Furthermore, the study examined the interaction effects across various subgroups, including age, gender, drinking status, smoking status, hypertension, diabetes, and dyslipidemia, utilizing multivariate logistic regression and Cox regression models. These interactions were evaluated using likelihood ratio tests.

In this study, we simultaneously constructed mediation effect models for the forward path (CCR→Depression→CKD) and the reverse path (CKD→Depression→CCR). Due to the limitation of the time factor, we investigated the mediating effect of depression in the bidirectional relationship between CCR-CKD and CKD-CCR based on the guidance of causal mediation analysis to validate the directionality of the causal path (33). Then, we constructed two models: a multifactorial logistic regression model with CCR as the exposure, other variables as confounders, and CKD as the outcome; and a multifactorial logistic regression model with CKD as the exposure, other variables as confounders, and CCR as the outcome. The mediator proportion was calculated by dividing the natural indirect effect by the total effect, utilizing the natural effects model estimation as proposed by VanderWeele (34).

In addition, we performed the following sensitivity analyses: (1) repeated all analyses using the complete dataset without any missing variables and without multiple interpolations; (2) computed E-values to assess the potential effect of unmeasured confounders in the mediation analyses; and (3) considering the possible influence of CES-D scale thresholds on the results, the results were tested for both thresholds of CESD ≥ 8 points and CESD ≥ 12 points.

## 3 Results

### 3.1 Baseline characteristics

In our final analysis, we included a total of 6,243 participants, with a mean age of 58.4 ± 8.8 years. The prevalence of CKD within the study population was 8.8%, and the mean depressive symptom score was 8.5 ± 6.3. Among the participants, 2,835(45.4%) were male and 3,408(54.6%) were female. We characterized the study participants based on CCR quartiles and found that the prevalence of nephropathy was 6.4% in the first quartile group of the CCR. With progressively higher CCR scores, the prevalence was 8.9% in the second quartile, 9.2% in the third quartile, and 10.6% in the fourth quartile. Lower CCR levels were associated with a higher incidence of CKD. In contrast to the increased incidence of CKD, all-cause mortality decreased significantly with increasing CCR. The all-cause mortality rate was 4.2% at the first quartile of the CCR and 0.9% at the fourth quartile of the CCR. In our study, we also characterized the CKD and non-CKD populations, and the CKD population had higher levels of CCR and depression than the non-CKD population (**Supplementary Table S2**). More detailed information about the basic characteristics of the population in this study is shown in **Table 1** and **Supplementary Table S2**.

**Table 1:** Baseline characterization of the CCR quartiles.

Level	Overall	Q1 (<6.84)	Q2 (<7.86)	Q3 (<9.12)	Q4 (≥9.12)	p
N	6243	1561	1562	1560	1560	
Age [mean (SD)]	58.4 (8.8)	61.2 (8.9)	59.4 (8.7)	57.5 (8.5)	55.6 (7.9)	<0.001
BMI [mean (SD)]	23.6 (3.9)	23.3 (4.2)	23.5 (3.9)	23.7 (3.5)	24.1 (3.7)	<0.001
CESD [mean (SD)]	8.5 (6.3)	9.7 (6.4)	8.9 (6.6)	8.1 (6.2)	7.4 (5.9)	<0.001
Gender (%)						<0.001
Female	3408 (54.6)	1192 (76.4)	976 (62.5)	723 (46.3)	517 (33.1)	
Male	2835 (45.4)	369 (23.6)	586 (37.5)	837 (53.7)	1043 (66.9)	
Drinking (%)						<0.001
No	4230 (67.8)	1232 (78.9)	1141 (73.0)	1000 (64.1)	857 (54.9)	
Yes	2013 (32.2)	329 (21.1)	421 (27.0)	560 (35.9)	703 (45.1)	
Smoking (%)						<0.001
Non-smoker	3887 (62.3)	1167 (74.8)	1035 (66.3)	888 (57.0)	797 (51.1)	
Ex-smoker	496 (7.9)	72 (4.6)	92 (5.9)	146 (9.4)	186 (11.9)	
Smoker	1858 (29.8)	322 (20.6)	435 (27.8)	525 (33.7)	576 (36.9)	
Hypertension (%)						0.236
No	3378 (58.7)	818 (56.6)	848 (58.4)	865 (60.0)	847 (59.6)	
Yes	2381 (41.3)	627 (43.4)	605 (41.6)	576 (40.0)	573 (40.4)	
Diabetes (%)						0.007
No	5712 (92.1)	1458 (93.8)	1427 (91.9)	1427 (92.4)	1400 (90.4)	
Yes	488 (7.9)	97 (6.2)	125 (8.1)	118 (7.6)	148 (9.6)	
Dyslipidemia (%)						<0.001
No	4672 (74.8)	1246 (79.8)	1226 (78.5)	1185 (76.0)	1015 (65.1)	
Yes	1570 (25.2)	315 (20.2)	336 (21.5)	374 (24.0)	545 (34.9)	
Chronic Kidney Disease (%)						<0.001
No	5694 (91.2)	1461 (93.6)	1423 (91.1)	1416 (90.8)	1394 (89.4)	
Yes	549 (8.8)	100 (6.4)	139 (8.9)	144 (9.2)	166 (10.6)	
Depression (%)						<0.001
N	3846 (61.6)	827 (53.0)	935 (59.9)	1004 (64.4)	1080 (69.2)	
Y	2397 (38.4)	734 (47.0)	627 (40.1)	556 (35.6)	480 (30.8)	
All cause mortality (%)						<0.001
No	6101 (97.7)	1496 (95.8)	1525 (97.6)	1534 (98.3)	1546 (99.1)	
Yes	142 (2.3)	65 (4.2)	37 (2.4)	26 (1.7)	14 (0.9)	

### 3.2 Association analysis of CCR with depressive symptoms and the risk of CKD

**Table 2** elucidates the CCR-CKD, CCR-depression and depression-CKD association. The results of the one-way logistic regression model showed that CCR was significantly associated with CKD(*OR: 1.10, 95% CI: 1.06-1.14, p<0.001*). This association persisted as statistically significant even after adjusting for various confounding factors. In the multivariate logistic regression analysis, Model 2 adjusted for age, gender, and BMI with an OR of 1.13(*95%CI:1.09-1.18, p<0.001*). Model 3, which further adjusted for alcohol consumption and smoking based on Model 2, also demonstrated an OR of 1.14(*95%CI:1.09-1.18, p<0.001*). Finally, Model 4, which incorporated adjustments for hypertension, diabetes and dyslipidemia based on Model 3, maintained an OR of 1.13 (*95% CI:1.09-1.18, p<0.001*). In addition, CCR was stratified into quartiles, revealing significant differences in the risk of CKD across varying CCR levels. Notably, individuals in the fourth quartile of CCR exhibited the highest risk of CKD, a finding that was particularly pronounced in Model 3(*OR: 2.34, 95%CI: 1.77-3.12, p<0.001*). This is consistent with the study by Hyun YY et al. A nationwide prospective cohort study conducted in Korea showed that higher CCR levels had lower eGFR values (35). And in our study, eGFR values represent to some extent CKD.Univariate logistic regression analysis indicated an association between CCR and depression, with an OR of 0.88(*95% CI: 0.86-0.91, p<0.001*) for the relationship between CCR and depression. This association persisted even after adjusting for sociodemographic, lifestyle, and health status variables, with an OR of 0.94(*95%CI: 0.91-0.96, p<0.001*). Lower CCR values were correlated with an increased risk of depression. The covariate adjustments in Model 3 and Model 4 were largely consistent. The findings were similar to those of Liu F et al, Zhu Y et al (19, 36). Depression is common in the CKD population and is associated with a significant risk of adverse outcomes (37). The study accounted for CCR while constructing logistic regression models to assess the risk of depression and CKD. In the univariate logistic regression model, depression was found to have a negative effect on CKD(*OR: 1.05, 95% CI: 1.04-1.07, p<0.001*). After controlling for confounding variables, the risk of CKD in individuals with depression was found to be 1.87 times greater than in those without depression(*OR: 1.87, 95%CI: 1.56-2.24, p<0.001*).

**Table 2:** CCR→Depression→CKD: Multivariate logistic regression models for CCR, depressive symptoms, and CKD.

Variable	Model 1	Model 2	Model 3	Model 4
	OR (95% CI)	OR(95% CI)	OR (95% CI)	OR (95% CI)
CCR and CKD				
CCR (continuous)	1.10 (1.06, 1.14)	1.13 (1.09, 1.18)	1.14 (1.09, 1.18)	1.13 (1.09, 1.18)
CCR (categorical)				
Quartile 1	1 [Reference]	1 [Reference]	1 [Reference]	1 [Reference]
Quartile 2	1.43 (1.09, 1.87)	1.56 (1.19, 2.05)	1.56 (1.19, 2.05)	1.54 (1.18, 2.03)
Quartile 3	1.49 (1.14, 1.94)	1.78 (1.35, 2.35)	1.80 (1.36, 2.38)	1.77 (1.34, 2.34)
Quartile 4	1.74 (1.35, 2.26)	2.29 (1.73, 3.05)	2.34 (1.77, 3.12)	2.26 (1.70, 3.03)
*P* for trend	<0.001	<0.001	<0.001	<0.001
CCR and depressive symptoms				
CCR (continuous)	0.88 (0.86, 0.91)	0.94 (0.91, 0.96)	0.94 (0.91, 0.96)	0.94 (0.91, 0.96)
CCR (categorical)				
Quartile 1	1 [Reference]	1 [Reference]	1 [Reference]	1 [Reference]
Quartile 2	0.76 (0.66, 0.87)	0.83 (0.72, 0.96)	0.84 (0.72, 0.97)	0.83 (0.72, 0.96)
Quartile 3	0.62 (0.54, 0.72)	0.78 (0.67, 0.90)	0.78 (0.67, 0.90)	0.77 (0.66, 0.90)
Quartile 4	0.50 (0.43, 0.58)	0.69 (0.59, 0.81)	0.70 (0.59, 0.82)	0.69 (0.59, 0.81)
*P* for trend	<0.001	<0.001	<0.001	<0.001
CESD/Depression and CKD (controlling for CCR)				
CESD,CCR (continuous)	1.05 (1.04, 1.07)	1.05 (1.04, 1.06)	1.05 (1.03, 1.06)	1.05 (1.03, 1.06)
Depression,CCR (categorical)				
No (≤ 10 scores)	1 [Reference]	1 [Reference]	1 [Reference]	1 [Reference]
Yes (> 10 scores)	1.96 (1.64, 2.34)	1.93 (1.61, 2.31)	1.90 (1.58, 2.28)	1.87 (1.56, 2.24)
*P* for trend	<0.001	<0.001	<0.001	<0.001

In addition, we also explored the association with CKD as exposure, other variables as confounders, and CCR as outcome. The results of the study, as shown in **Table 3**, showed a strong association between CKD and CCR (*p<0.001*). Although there is a paucity of studies with CKD as exposure and CCR as outcome. We also analyzed the inverse association of CKD as an exposure to depression, consistent with previous studies that found depression to be more common in the CKD population. For subsequent analysis of bidirectional mediating effects, in the context of controlling for CKD, we also explored the effect of depression on CCR to control for confounding.

**Table 3:** CKD→Depression→CCR: Multivariate logistic regression models for CCR, depressive symptoms, and CKD.

Variable	Model 1	Model 2	Model 3	Model 4
	OR (95% CI)	OR(95% CI)	OR (95% CI)	OR (95% CI)
CKD and CCR (categorical)				
No	1 [Reference]	1 [Reference]	1 [Reference]	1 [Reference]
Yes	1.55 (1.24, 1.95)	1.79 (1.42, 2.28)	1.80 (1.43, 2.30)	1.77 (1.39, 2.26)
*P* for trend	<0.001	<0.001	<0.001	<0.001
CKD and depressive symptoms				
No	1 [Reference]	1 [Reference]	1 [Reference]	1 [Reference]
Yes	1.85 (1.55, 2.20)	1.85 (1.55, 2.22)	1.82 (1.52, 2.18)	1.80 (1.50, 2.16)
*P* for trend	<0.001	<0.001	<0.001	<0.001
Depression and CCR (categorical) (controlling for CKD)				
Depression, CCR (categorical)				
No (≤ 10 scores)	1 [Reference]	1 [Reference]	1 [Reference]	1 [Reference]
Yes (> 10 scores)	0.61 (0.54, 0.68)	0.74 (0.65, 0.84)	0.76 (0.67, 0.86)	0.76 (0.67, 0.86)
*P* for trend	<0.001	<0.001	<0.001	<0.001

### 3.3 Association analysis of CCR and all-cause mortality

**Figure 2:**
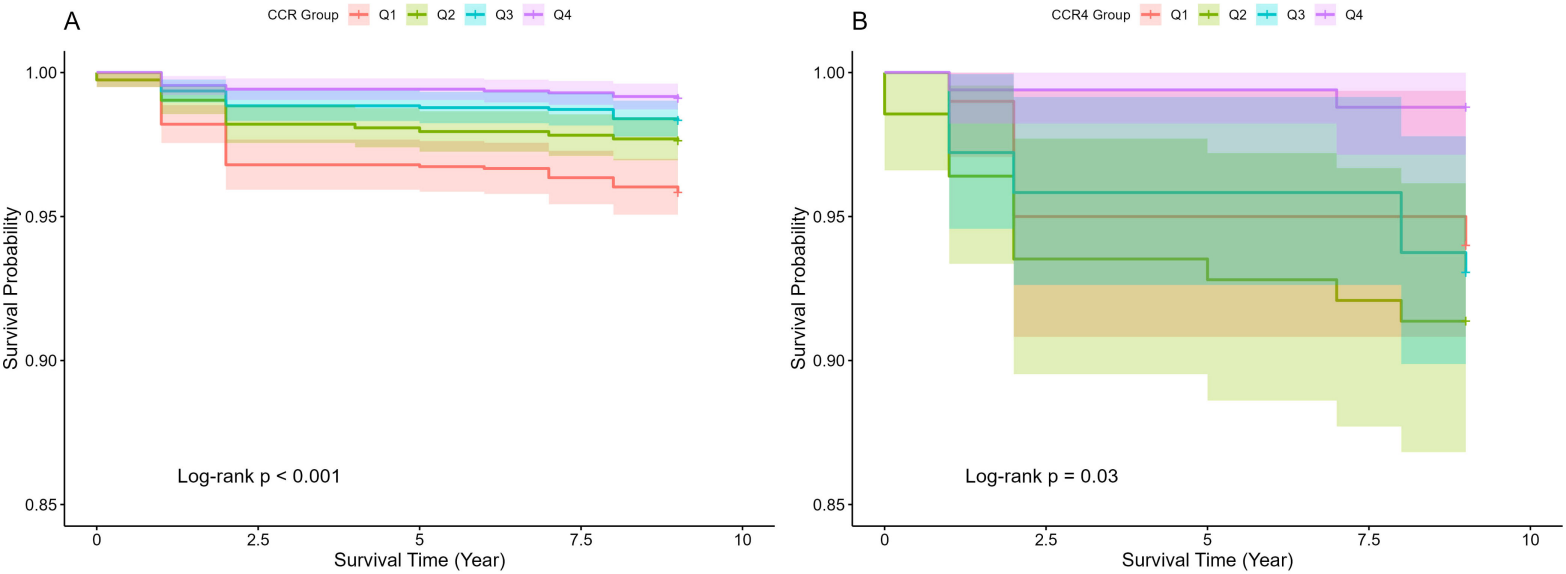
Overall survival curves of CCR for general populations **(A)** and CKD populations **(B)**.

The CCR is an emerging marker that has been increasingly studied in relation to adverse health outcomes (38). Several studies have shown that lower levels of CCR are associated with higher all-cause mortality (12, 13, 39). In this study, the correlation between CCR and all-cause mortality was further verified using Kaplan-Meier curves, which showed that survival rates were not the same in populations with different CCR levels, generally presenting a lower risk of death with higher CCR levels (*p<0.05*), as illustrated in **Figure 2**. The risk of mortality was notably higher in the CKD cohort (**Figure 2B**), compared to the general population (**Figure 2A**), aligning with established trends. A multivariate cox proportional hazards regression model was employed to evaluate the association between CCR and all-cause mortality, as detailed in **Table 4**. The analysis revealed that as CCR levels increased, the risk of mortality decreased within the study population. Model 4 was adjusted for the effects of the additional variables including age, gender, drinking, and hypertension, and yielded a HR of 0.85(*95% CI: 0.75-0.97, p=0.012*). The fourth quartile of the CCR represents a range of elevated CCR levels, and the performance of the model adjusted for the rest of the variables indicates that high CCR levels are associated with a reduced risk of mortality(*HR: 0.35, 95% CI: 0.18-0.67, p=0.001*).

**Table 4:** Multivariate cox proportional hazards regression models for CCR and all-cause mortality.

Variable	Model 1	Model 2	Model 3	Model 4
	HR (95% CI)	HR(95% CI)	HR (95% CI)	HR (95% CI)
CCR (continuous)	0.76 (0.69, 0.84)	0.84 (0.75, 0.94)	0.84 (0.75, 0.94)	0.83 (0.74, 0.93)
CCR (categorical)				
Quartile 1	1 [Reference]	1 [Reference]	1 [Reference]	1 [Reference]
Quartile 2	0.56 (0.38, 0.84)	0.63 (0.40, 0.91)	0.61 (0.41, 0.92)	0.59 (0.39, 0.90)
Quartile 3	0.39 (0.25, 0.62)	0.48 (0.30, 0.77)	0.47 (0.29, 0.77)	0.46 (0.29, 0.74)
Quartile 4	0.21 (0.12, 0.38)	0.34 (0.18, 0.62)	0.34 (0.18, 0.62)	0.31 (0.17, 0.58)
*P* for trend	<0.001	0.001	0.001	<0.001

### 3.4 Restricted cubic spline to explore the association between CCR and the risk of CKD, depressive symptoms, and all-cause mortality

**Figure 3:**
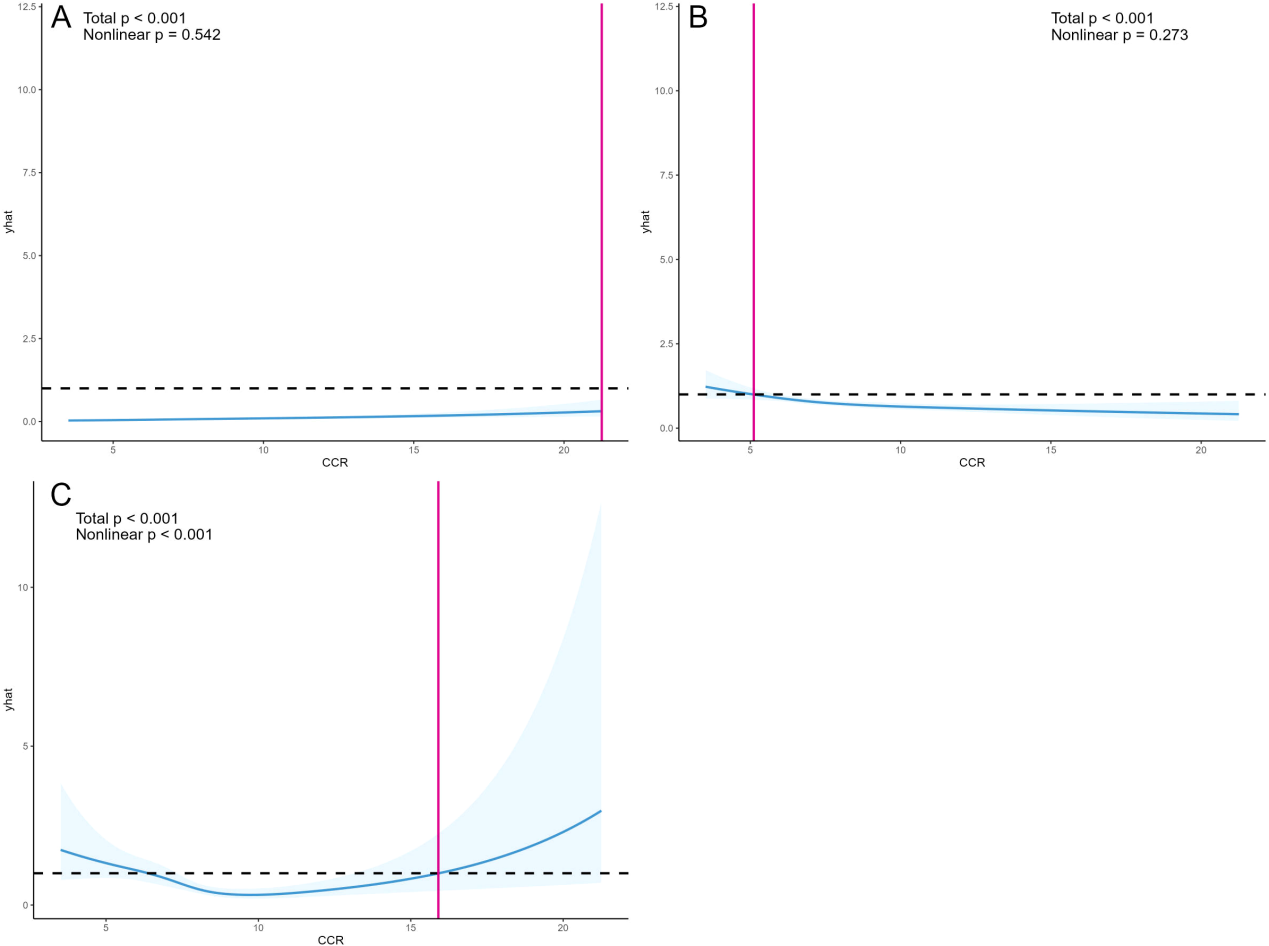
OR and 95% CI for CCR and the risk of CKD **(A)**, OR and 95% CI for CCR and depressive symptoms **(B)**, and HR and 95% CI for CCR and all-cause mortality **(C)**. Restricted cubic splines were constructed based on Model 4, adjusting for age, gender, BMI, alcohol consumption, smoking status, hypertension, diabetes, and dyslipidemia. The solid blue line represents the multivariate-adjusted HR/OR, while the blue shaded area represents the 95% CI.

Utilizing Model 4, the study employed restricted cubic splines to determine the linearity or non-linearity of relationships between variables, with findings presented in **Figure 3**. The analysis revealed a linear relationship between CCR and CKD, as indicated by a nonlinear p-value of 0.542 (*p>0.05*), and a similar linear relationship with depression, with a nonlinear p-value of 0.273 (*p>0.05*). Conversely, CCR and all-cause mortality exhibited a significant nonlinear correlation (*p<0.05*), characterized by a “U” curve.

### 3.5 Subgroup analysis

**Figure 4:**
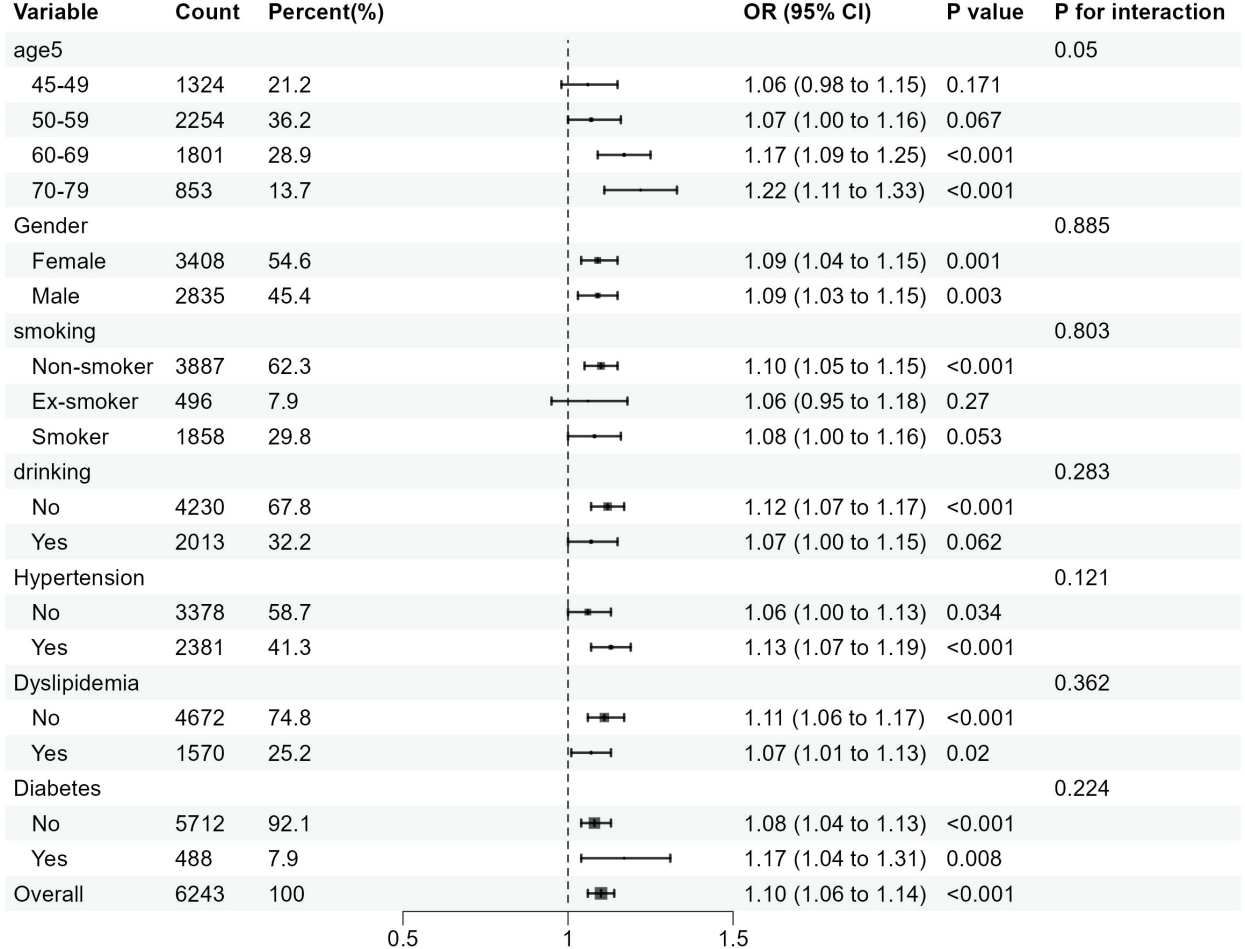
Subgroup analysis of the association between CCR and the risk of CKD. OR is the odds ratio and 95% CI is the 95% confidence interval.

**Figure 5:**
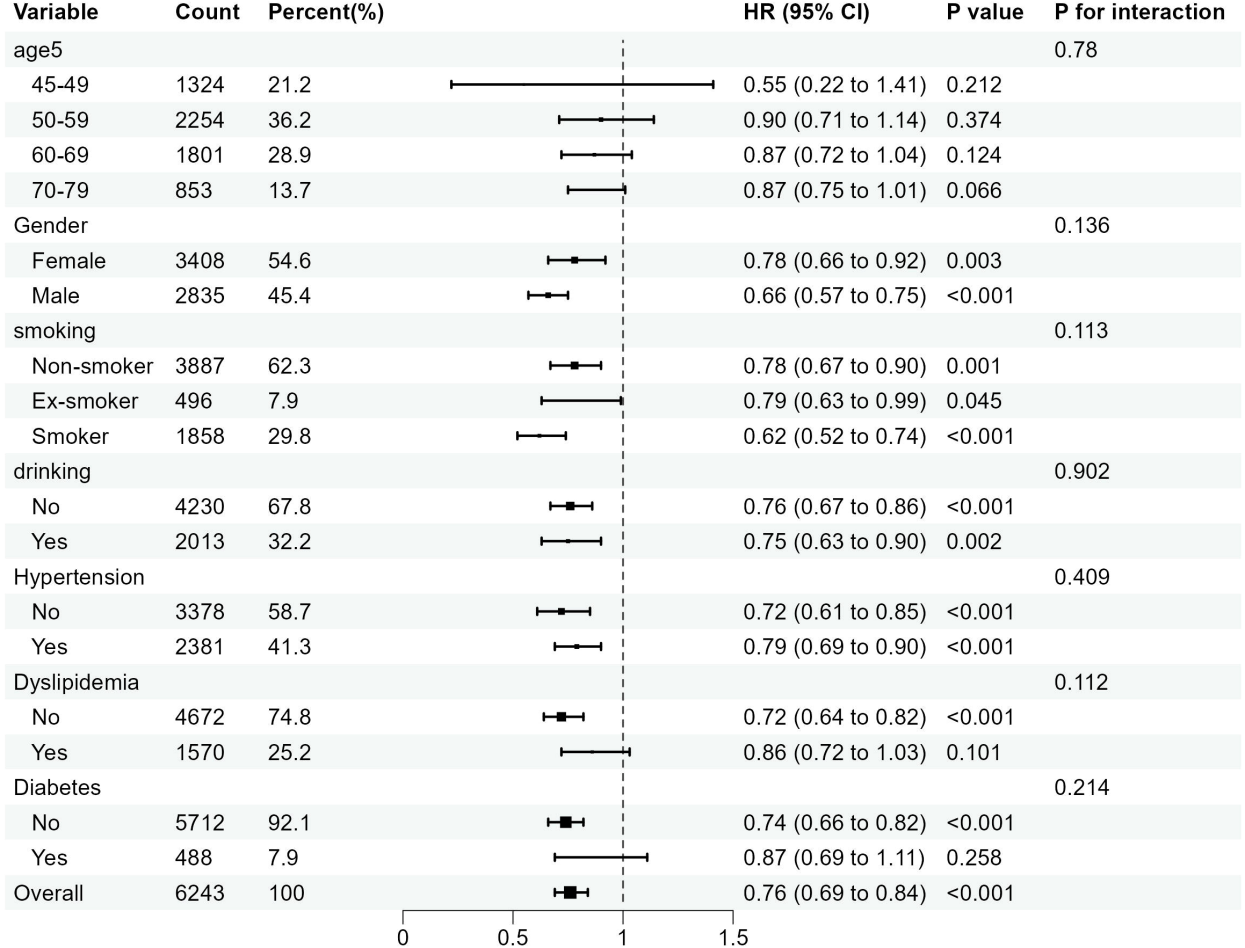
Subgroup analysis of the association between the CCR and all-cause mortality. OR is the odds ratio and 95% CI is the 95% confidence interval.

In this study, subgroup analyses focusing on CCR-CKD and CCR-All-cause mortality were conducted separately, with results depicted in **Figures 4** and **5**. These subgroup analyses were consistent with the overall analysis. The association between the CCR and CKD was more pronounced in individuals over 60 years of age, non-smokers, and non-alcohol drinkers. The association between the CCR and all-cause mortality was more pronounced among individuals with normal lipids and those without diabetes. The association between CCR values and variables such as age, gender, alcohol consumption, smoking status, hypertension, diabetes, and dyslipidemia did not have an interaction (*p>0.05*).

### 3.6 Mediation analysis of depressive symptoms in the association between CCR and the risk of CKD

**Figure 6:**
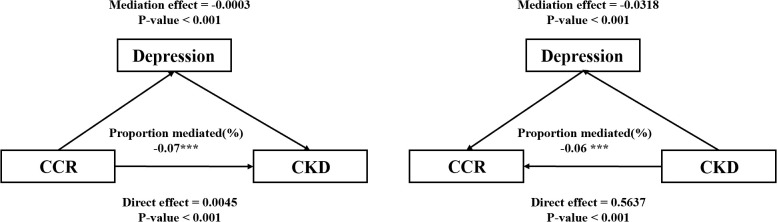
Mediating effect of depressive symptoms in CCR and the risk of CKD. *** represents p-values less than 0.001.

The study explored the bidirectional mediating utility of depression in the relationship between CCR and CKD based on model 4, and the results of the analysis are shown in **Figure 6**. The results suggest that depression mediates the association between CCR and CKD, although the mediating effect may be relatively small. After excluding as much as possible the effects of confounding factors such as age, gender, and chronic diseases, depression exerted a weak negative mediating effect on the effect of CCR on CKD (*mediation effect=-0.0003, 95%CI:-0.0005-0.0000, p<0.001*), and an even more pronounced negative mediating effect on the reverse effect of CKD on CCR (*mediation effect=-0.0318, 95%CI:-0.0547-0.0200, p<0.001*). Although the Nagelkerke R^2^ was lower and the explanatory power of the variable was weaker, we believe that this suggests that CKD may indirectly lead to a decrease in CCR levels by exacerbating depression. The relevant computational information for the bidirectional mediated effects model of this study is presented in **Supplementary Table S3**.

### 3.7 Sensitivity analysis

To ensure the accuracy of the funding of the study, three methods were used in this study for sensitivity analysis. The study produced a complete dataset without any missing variables, a post-interpolated dataset with depression judged by a CESD score of ≥8, and a post-interpolated dataset with depression judged by a CESD score of ≥12. Multifactor logistic regression analyses of CCR- CKD, CCR-depressive, and depressive-CKD were performed for each of the three datasets (all based on model 4), as well as multifactor cox proportional risk regression modeling of CCR and all-cause mortality (based on model 4). The results of the data analyses were consistent with previous data analyses. In addition, the study used E-values to assess sensitivity analyses for the likelihood of unmeasured confounders, and the results are supplemented with **Supplementary Table S4**, **Supplementary Table S5** and **Supplementary Table S6**.

## 4 Discussion

This study utilized data from a nationally representative sample of 6,243 Chinese middle-aged and elderly adults to investigate the relationship between CCR, depression, CKD and all-cause mortality, and the potential bidirectional mediating effect of depression in the association between CCR and CKD. Our findings indicated that greater CCR levels were positively correlated with an increased risk of CKD while being negatively related to depression and all-cause mortality. Depression plays a bidirectional negative mediating role in the association between CCR and CKD, albeit a relatively weak one. Subgroup and sensitivity analyses affirmed the robustness of these results. To our knowledge, this is the inaugural study to concurrently investigate the association of CCR with CKD and all-cause mortality, along with the bidirectional mediating effect of depression on the association between CCR and CKD, within a middle-aged and elderly Chinese population.

In this study, our results suggest an association between CCR and CKD. Previous studies have shown that CCR can be used as a biopredictive marker for CKD with higher sensitivity and specificity in assessing renal function abnormalities (35). Creatinine, as a metabolite of muscle tissue, is mainly eliminated from the body through the kidneys. However, there is a certain lag in creatinine measurement, which usually fails to detect CKD in a timely and accurate manner, whereas cystatin C, a low-molecular-weight protein, is completely dependent on glomerular reabsorption and degradation (9), which can make up for the limitations of creatinine measurement to a certain extent (40). Therefore, CCR can synthesize the advantages of both and help to detect the occurrence and progression of CKD in time (41). Current studies have focused on the association of CCR with mortality or all-cause mortality in CKD and other diseases, and the relationship between CCR and CKD is not clear enough. Nevertheless, we still found some similarities with our study. A study comparing creatinine, cystatin C, and iohexol clearance measurements for the identification of persistent renal function found a correlation between changes in CCR and muscle loss (*R=0.61, 95% CI:0.50-0.72*) *(* 42). The progression of CKD inevitably presents with muscle atrophy, and early detection of muscle loss is crucial for the detection of CKD (43). Furthermore, a case report describes the development of CKD accompanied by elevated levels of CCR (44). This is consistent with our findings using CKD as an exposure factor, where a higher risk of CKD was associated with higher CCR levels. Since we used cross-sectional data, we could not explore the causal relationship between CCR and CKD. However, exploring the association between CCR and CKD is an important addition to further exploring the possibility that CCR may be a valid predictive biomarker of CKD risk in the future.

A meta-analysis has indicated that depression is more prevalent among CKD patients and that elevated depression are correlated with a heightened risk of CKD (15). In this study, the prevalence of depression among participants with CKD was higher compared to those without CKD, aligning with findings from previous research. However, a retrospective cross-sectional study has demonstrated an association between low CCR levels and elevated depression (19). We also analyzed the CCR-depression and depression-CKD association in our study, validating previous findings. Due to the limitation of time factor, we used bidirectional mediated effects analysis to establish two models for the forward path (CCR→Depression→CKD) and the reverse path (CKD→Depression→CCR). We further explored the effect of depression between CCR-CKD and CKD-CCR based on Model 4. Model 4 controlled the interference of confounders to some extent and satisfied the hypothesis of mediation analysis. The findings indicated that the negative effect of depression was stronger in the association of CKD-CCR than in the association of CCR-CKD. Despite the weaker explanatory strength of the model, we believe this suggests that CKD may contribute to death by exacerbating depression and indirectly leading to lower levels of CCR. Specifically, higher CKD risk was associated with higher CCR levels, but depression may have attenuated this effect. This is likely because patients with depression are often accompanied by increased levels of inflammation, and inflammatory cells are associated with increased levels of cystatin C, leading to underestimated CCR levels. In addition, depressed populations are inextricably linked to lower creatinine due to decreased activity levels. Reduced creatinine levels also lead to underestimation of CCR levels. CKD patients are a high-prevalence group for depression, which not only affects the subjective quality of life of patients but also interferes with biomarkers for monitoring and management of CKD. Therefore, the mental health of CKD patients needs to be emphasized. Although the CCR is effective in predicting CKD, its use in the depressed middle-aged and elderly population requires additional attention to the negative effects of depression.

Our findings corroborate the established association between CCR and all-cause mortality. A prospective cohort study conducted in Korea concluded that elevated levels of CCR were linked to reduced all-cause mortality (35). Our findings align with these previous studies, confirming similar conclusions. In this study, we also generated the kaplan-meier survival curves of CCR across the entire cohort and specifically for all-cause mortality in individuals with CKD. The analysis revealed that higher CCR levels correspond to increased survival rates regardless of CKD. Moreover, the restricted cubic spline analysis indicated that the relationship between CCR and all-cause mortality is nonlinear, exhibiting a “U”-shaped curve.

This study possesses both strengths and limitations. One of the primary advantages is the utilization of data from a nationally representative sample of middle-aged and elderly people in China, a country characterized by its large population and aging demographic. The results of this study are of great practical significance for the health management and intervention of CKD in middle-aged and elderly people. The employment of a large sample of dataset, the development of multiple models and the application of sensitivity analyses reinforced the robustness of the study results. However, our study also has certain limitations. Firstly, the definition of CKD within the CHARLS database relies on participants’ self-reporting rather than verified medical diagnosis. Although we simultaneously examined participants’ CKD medication intake as well as calculated participants’ eGFR values, there was still selective bias or measurement bias. Second, due to the lack of detailed dietary intake and systemic inflammatory marker data in CHARLS, this study failed to include dietary composition and inflammatory indicators, which may constitute potential residual confounding. The study relied on cross-sectional data precludes to determine causality. Even though we explored the bidirectional mediating effect of depression in the CCR-CKD and CKD-CCR associations, we were unable to confirm causality between the other variables. As we utilized data from middle-aged and elderly individuals aged 45 and 79, the findings of this study cannot be extrapolated to younger populations for application.

## 5 Conclusion

Overall, We analyzed the data based on the CHARLS in 2011 and 2015. The results showed that higher levels of CCR were associated with a higher risk of CKD and with lower all-cause mortality, and CCR could be a biomarker for early diagnosis of CKD. CCR was negatively correlated with depression, and depression may have a negative effect on the association of CKD and CCR, suggesting that CCR has an underestimated risk for predicting and recognizing CKD in depressed populations. Mental health factors are important in risk prediction and comprehensive management of CKD. Future studies may further explore the mechanism of depression in the progression of CKD and the potential causal relationship with biomarkers through prospective cohort studies and incorporate psychological intervention strategies to optimize the prevention, monitoring, treatment, and prognostic management of CKD.

## Data Availability

Publicly available datasets were analyzed in this study. This data can be found here: China Health and Retirement Longitudinal Study (CHARLS) online datasets (https://charls.pku.edu.cn/).
