# Supplementary material for: Bidirectional relationship between serum creatinine to cystatin C ratio and chronic kidney disease: a mediation analysis of depression in a national aging cohort
Source: Front Psychiatry. 2025 May 16;16:1554695. doi: 10.3389/fpsyt.2025.1554695 (PMC12123441; doi:10.3389/fpsyt.2025.1554695)
Supplement: Supplementary file 1 [file Supplementaryfile1.docx]

Supplementary Material

# Supplementary Information

## Supplementary Figures

**Figure S1. Missing data**

**
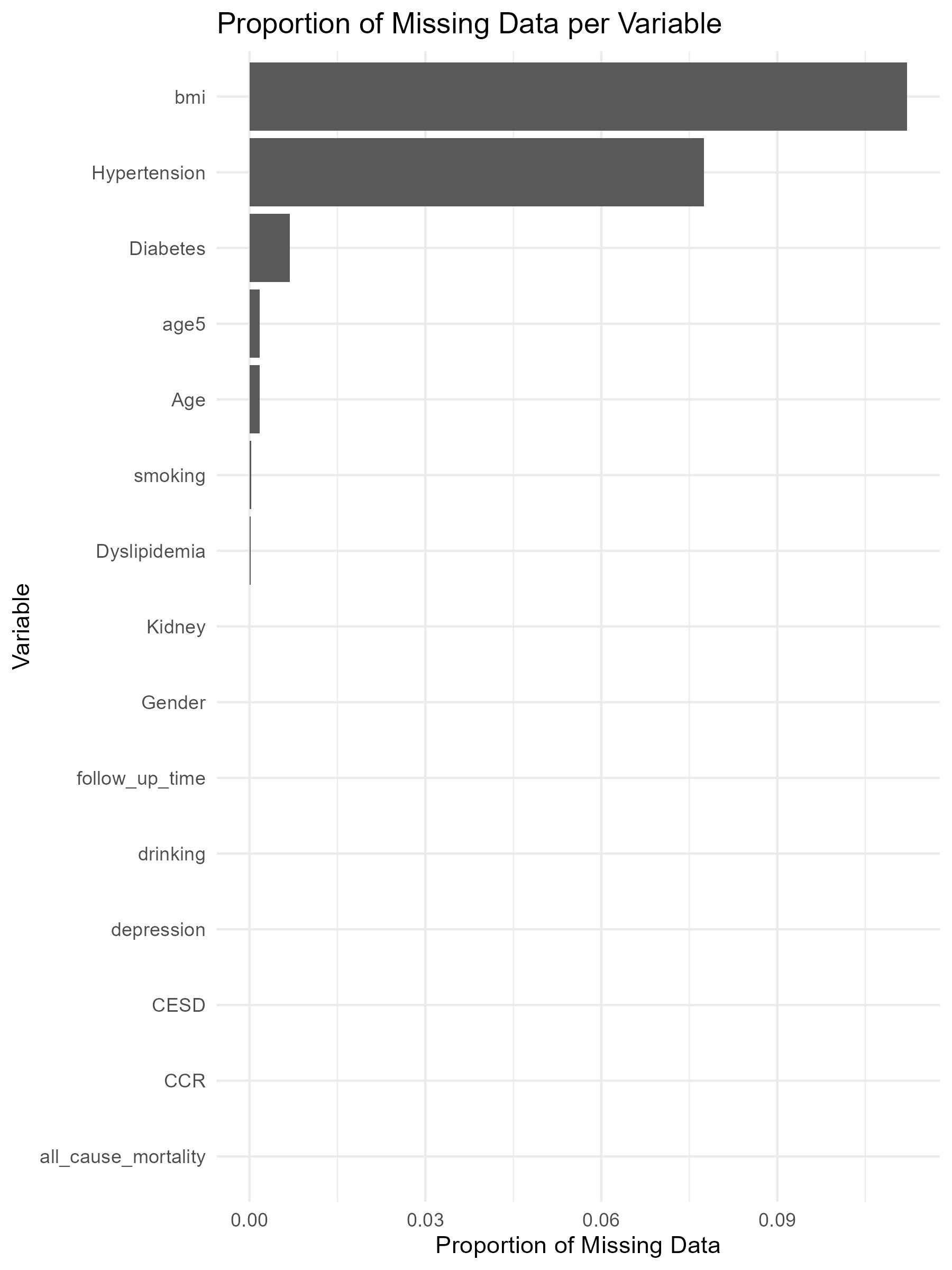
**

**Figure S2. Pattern (Missing data)**

**
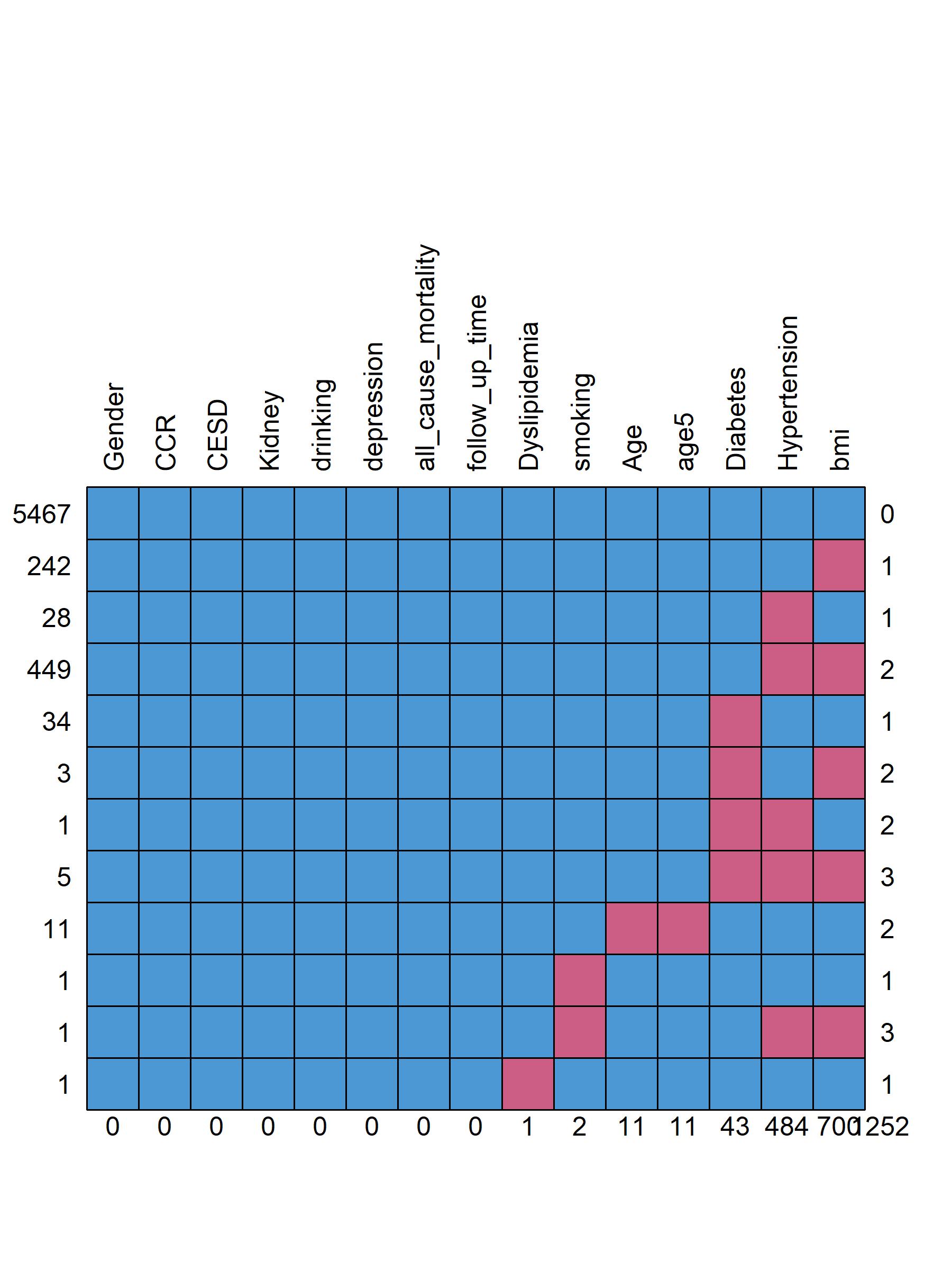
**

## Supplementary Tables

**Table S1.** Definitions and classification of the covariates

| Variables | Types | Definitions and classification |
| --- | --- | --- |
| Age | Continuous/ Categories | With categories: 45-49, 50-59, 60-69, 70-79 |
| bmi | Continuous | Weight in kilograms divided by height in meters squared (kg/m2) |
| CESD | Continuous | Depressive symptoms were assessed using the 10-item Center for Epidemiological Survey Depression Scale |
| Gender | Categories | Male; female |
| Drinking | Categories | No: None of these; Yes: Drink more than once a month/ Drink but less than once a month |
| Smoking | Categories | Non-smoker: No smoking; Ex-smoker: Used to smoke but quit; Smoker: Still smoking |
| Hypertension | Categories | No: Self-reported no hypertension and systolic blood pressure less than 140 mm Hg and diastolic blood pressure 90 mm Hg and no medication; Yes: not matching the former |
| Diabetes | Categories | No: Self-reported no diabetes or high blood sugar and fasting blood glucose less than 6.5 mmol/L and no medication; Yes: not matching the former |
| Dyslipidemia | Categories | No: Self-reported no dyslipidemia and total cholesterol less than 6.2 mmol/L and triglyceride less than 2.26 mmol/L and low-density lipoprotein less than 4.14 mmol/L and no medication; Yes: not matching the former |
| Kidney | Categories | Yes: Self-reported chronic kidney disease or eGFR less than 60 ml/min/1.73 m^2^; No: not matching the former |
| depression | Categories | No: CESD scores below 10; Yes: CESD scores greater than or equal to 10 |
| All cause mortality | Categories | Yes: The exact date of death documented on a death certificate or within a medical record; No: not matching the former |

**Table S2.** Baseline characterization of the CKD

| level | Overall | Non-CKD | CKD | p |
| --- | --- | --- | --- | --- |
| N | 6243 | 5694 | 549 |  |
| Age (mean (SD)) | 58.4 (8.8) | 58.2 (8.7) | 61.0 (9.3) | <0.001 |
| BMI (mean (SD)) | 23.6 (3.9) | 23.7 (3.8) | 23.5 (4.3) | <0.001 |
| CESD (mean (SD)) | 8.5 (6.3) | 8.4 (6.2) | 10.4 (7.2) | <0.001 |
| CCR (mean (SD)) | 8.1 (2.1) | 8.1 (2.0) | 8.5 (2.5) | <0.001 |
| Gender (%) |  |  |  |  |
| Female | 3408 (54.6) | 3132 ( 55.0) | 276 ( 50.3) | 0.037 |
| Male | 2835 (45.4) | 2562 ( 45.0) | 273 ( 49.7) |  |
| drinking (%) |  |  |  |  |
| No | 4230 (67.8) | 3838 ( 67.4) | 392 ( 71.4) | 0.062 |
| Yes | 2013 (32.2) | 1856 ( 32.6) | 157 ( 28.6) |  |
| smoking (%) |  |  |  |  |
| Non-smoker | 3888 (62.3) | 3579 ( 62.9) | 309 ( 56.3) | 0.001 |
| Ex-smoker | 496 ( 7.9) | 434 ( 7.6) | 62 ( 11.3) |  |
| Smoker | 1859 (29.8) | 1681 ( 29.5) | 178 ( 32.4) |  |
| Hypertension (%) |  |  |  |  |
| No | 3684 (59.0) | 3419 ( 60.0) | 265 ( 48.3) | <0.001 |
| Yes | 2559 (41.0) | 2275 ( 40.0) | 284 ( 51.7) |  |
| Diabetes (%) |  |  |  |  |
| No | 5754 (92.2) | 5261 ( 92.4) | 493 ( 89.8) | 0.038 |
| Yes | 489 ( 7.8) | 433 ( 7.6) | 56 ( 10.2) |  |
| Dyslipidemia (%) |  |  |  |  |
| No | 4672 (74.8) | 4277 ( 75.1) | 395 ( 71.9) | 0.114 |
| Yes | 1571 (25.2) | 1417 ( 24.9) | 154 ( 28.1) |  |
| CCR4 (%) |  |  |  |  |
| Q1 | 1561 (25.0) | 1461 ( 25.7) | 100 ( 18.2) | <0.001 |
| Q2 | 1562 (25.0) | 1423 ( 25.0) | 139 ( 25.3) |  |
| Q3 | 1560 (25.0) | 1416 ( 24.9) | 144 ( 26.2) |  |
| Q4 | 1560 (25.0) | 1394 ( 24.5) | 166 ( 30.2) |  |
| depression (%) |  |  |  |  |
| N | 3846 (61.6) | 3583 ( 62.9) | 263 ( 47.9) | <0.001 |
| Y | 2397 (38.4) | 2111 ( 37.1) | 286 ( 52.1) |  |

**Table S3.** The outcome of two-way mediation effect analysis

| Classes | CCR→Depression→CKD pathway | CKD→Depression→CCR pathway |
| --- | --- | --- |
| Mediation effect | -0.0003*** | -0.0318*** |
| Direct effect | 0.0045*** | 0.5637*** |
| Total effect | 0.0043*** | 0.5319*** |
| Proportion mediated | -0.07*** | -0.06*** |
| Cohen's d | -0.026 | 0.29 |
| Nagelkerke R^2^ | 0.04 | 0.05 |

*** represents p-values less than 0.001.

**Table S4.** Sensitivity analysis: complete dataset without any missing variables

| Classes | Variables | E-value |
| --- | --- | --- |
| CCR and CKD | Q1 | Ref |
|  | Q2 | 3.37 |
|  | Q3 | 3.24 |
|  | Q4 | 1.94 |
| CCR and Depression | Q1 | Ref |
|  | Q2 | 0.48 |
|  | Q3 | 0.47 |
|  | Q4 | 0.46 |
| Depression and CKD | No | Ref |
|  | Yes | 2.91 |
| CCR and All-cause mortality | Q1 | Ref |
|  | Q2 | 2.67 |
|  | Q3 | 4.19 |
|  | Q4 | 5.41 |

**Table S5.** Sensitivity analysis: potential effects of unmeasured confounders in mediated analysis

| Classes | E-value |
| --- | --- |
| CCR→Depression→CKD pathway | 1.02 |
| CKD→Depression→CCR pathway | 1.20 |

**Table S6.** Sensitivity analysis: dataset with two depression thresholds (after multiple interpolation)

| Classes | Variables | E-value |
| --- | --- | --- |
| CCR and Depression（CESD≥8分） | Q1 | Ref |
|  | Q2 | 0.48 |
|  | Q3 | 0.48 |
|  | Q4 | 0.46 |
| Depression and CKD（CESD≥8分） | No | Ref |
|  | Yes | 0.46 |
| CCR and Depression（CESD≥12分） | Q1 | Ref |
|  | Q2 | 0.48 |
|  | Q3 | 0.47 |
|  | Q4 | 0.46 |
| Depression and CKD（CESD≥8分） | No | Ref |
|  | Yes | 0.5 |
